# Lymphotoxin-Alpha Gene Polymorphism +252A/G (rs909253, A/G) Is Associated with Susceptibility to Chronic Periodontitis: A Pilot Study

**DOI:** 10.5402/2012/617245

**Published:** 2012-09-25

**Authors:** Daniel Fernando Pereira Vasconcelos, Marco Antônio Dias da Silva, Marcelo Rocha Marques, Rui Barbosa de Brito Júnior, Any Carolina Cardoso Guimarães Vasconcelos, Silvana Pereira Barros

**Affiliations:** ^1^Division of Histology and Embryology, School of Biomedicine, Federal University of Piauí, 64049-550 Parnaíba, PI, Brazil; ^2^Division of Histology and Embryology, Federal University of Campina Grande, 58109-990 Patos, PB, Brazil; ^3^Department of Morphology, Division of Histology, School of Dentistry at Piracicaba, University of Campinas, 13083-970 Piracicaba, SP, Brazil; ^4^Department of Morphology, Division of Histology, Institute São Leopoldo Mandic, 13045-610 Campinas, SP, Brazil; ^5^Division of Histology and Embryology, School of Physiotherapy, Federal University of Piauí, 64049-550 Parnaíba, PI, Brazil; ^6^Center for Oral and Systemic Diseases, Department of Periodontology, UNC School of Dentistry, University of North Carolina at Chapel Hill, Chapel Hill, NC 27599-7450, USA

## Abstract

*Background*. Periodontal disease leading to clinical findings such as increased periodontal probing depth involves a complex interaction between invading pathogenic microorganisms and the patient's immune system. Lymphotoxin alpha (LT-**α**) is a potent multifunctional immune modulator that contributes toward susceptibility to immune regulation disorders, including periodontal disease. *Objective*. In this study, we tested the hypothesis that chronic periodontitis (CP) is associated with polymorphisms of the LT-**α** gene. *Materials and Methods*. A total of 126 subjects, 44 healthy subjects, and 82 subjects with CP, were evaluated for periodontal disease by measuring clinical attachment loss and separation. Samples of epithelial cells were obtained for DNA analysis by scraping of the buccal mucosa. The LT-**α** gene was analyzed by polymerase chain reaction followed by endonuclease digestion with *Nco*I to analyze restriction fragment length polymorphisms. *Results*. The LT-**α** gene (+252A/G) polymorphism was associated with CP. LT-**α** allele frequencies were significantly different (*P* = 0.0019) between patients with CP and healthy individuals, with an odds ratio of 2.67 for patients with CP with the G allele. *Conclusions*. These findings suggest the LT-**α** gene genotype is a risk indicator for susceptibility to chronic periodontal disease in the Brazilian population studied.

## 1. Introduction

Periodontal disease is characterized by inflammatory destruction of tissues that support the tooth, leading to loss of epithelial attachment and destruction of the periodontal ligament, cementum, and alveolar bone, and eventually tooth loss [1].

 Etiological studies show that although bacterial infection causes periodontitis, a subset of individuals show severe disease in the absence of a large bacterial load, suggesting that these individuals mount an exaggerated response to a modest microbial challenge. Thus, the patient's immune response may play a critical role in periodontal disease pathogenesis and expression [1, 2]. This observation has led to interest in finding potential genetic markers of disease susceptibility, including polymorphisms in genes encoding key molecules of the host defense system, such as cytokines [3–5].

Lymphotoxin-*α* (LT-*α*), previously known as tumor necrosis factor (TNF)-*β*, is a proinflammatory cytokine encoded by the LT-*α* gene (HUGO Gene Nomenclature Committee, http://www.genenames.org/). LT-*α* plays a key role in orchestrating the inflammatory and immune responses involved in tissue destruction and bone resorption and has been suggested to be an important participant in the pathogenesis of periodontal disease [6, 7].

The LT-*α* gene contains several polymorphisms. Messer first reported the existence of a single nucleotide polymorphism (SNP) in the intron of LT-*α* at position 252 (G>A, designated rs909253, http://www.ncbi.nlm.nih.gov/snp/), which is associated with overexpression of LT-*α* [8]. The SNP rs 909253 was genotyped in the HapMap and selected as a haplotype-tagging SNP to capture common variations (minor allele frequency (MAF) > 0.05) across the TNF/LT-*α* gene region at the threshold of *r* ^2^ ≥ 0.8, based on the HapMap analysis [9].

The LT-*α* polymorphism has been the focus of several studies examining susceptibility genes in diseases of the immune system and has been associated with diseases such as rheumatoid arthritis [10], asthma [11], and cardiac diseases [12, 13]. However, only one study has examined the relationship between the LT-*α* (+252A/G) polymorphism and periodontitis, which indicated that the LT-*α* (+252A/A) genotype may be protective against CP in the Czech population [14]. In contrast, the studies by Ozaki et al. [12] and Ramasawmy et al. [15] found no association between the LT-*α* (+252A/A) genotype and protection from cardiac diseases.

Thus, the previous studies on disease association of the LT-*α* (+252A/G) polymorphism are ambiguous and inconclusive. Some of the discrepancies may result from lack of consideration of other risk factors of the disease, especially, differences in the lifestyle among the subjects as well as among ethnic groups [16], which may attenuate or strengthen the biological effects of the gene polymorphism. Another potential explanation for the discrepant findings is that a particular polymorphism may be a surrogate for other functional genetic polymorphisms in adjacent genes, the frequency of which may differ between populations. The aim of this study was to determine whether CP is associated with the *Nco*I polymorphism present in intron 1 (+252A/G) of the LT-*α* gene in a Brazilian population.

## 2. Materials and Methods

### 2.1. Selection of Subjects

The study population consisted of 126 Caucasians from the southeastern region of Brazil. The patients were referred to the Dental Clinics of the Faculty of Dentistry at Piracicaba, UNICAMP (approved by the Ethical Committee in Research at FOP/UNICAMP number 76/2003), and the study population included unrelated subjects aged >25 years (mean age, 44.6 years). The baseline clinical parameters for the subject population are presented in Table 1. All subjects were in good general health and had at least 20 teeth. The exclusion criteria for patient selection were as follows: smokers; diseases of the oral hard or soft tissues except caries and periodontal disease; use of orthodontic appliances; need for premedication for dental treatment; chronic usage of anti-inflammatory drugs; a history of diabetes, hepatitis, or HIV infection; immunosuppressive chemotherapy; history of any disease known to severely compromise immune function; current acute necrotizing ulcerative gingivitis; or current pregnancy or lactation. Medical and clinical information was obtained by a single examiner. Periodontal disease severity was diagnosed and classified on 1999 Consensus Classification of Periodontal Diseases [7], including physical examination, probing pocket depth, clinical attachment level (CAL), tooth mobility, gingival recession, and bleeding on probing. Measurements of probing depth and CAL were recorded at 6 sites around each tooth. Included subjects were divided into 2 groups.

*(A) Control Group (CG).* Subjects exhibited no signs of periodontal disease as determined by the absence of CAL, no sites with probing depth >3 mm, and less than 10% of sites with gingivitis (*n* = 44).

*(B) Chronic Periodontitis (CP).* Patients with teeth exhibiting >5 mm CAL, and at least 6 teeth exhibiting CAL, in at least 2 quadrants (*n* = 82).

### 2.2. Analysis of Genetic Polymorphisms

#### 2.2.1. Sampling

For sampling of epithelial buccal cells, individuals used a mouthwash with 5 mL of 3% glucose for 1 min. Following this, a sterile wooden spatula was used to scrape the oral mucosa. The tip of the spatula was then stirred in the individual's mouthwash solution. The solution was centrifuged at 2000 rpm for 10 min to pellet buccal epithelial cells, and the supernatant was discarded. The cell pellet was resuspended in 500 *μ*L of extraction buffer (10 mM Tris-HCl (pH 7.8), 5 mM EDTA, 0.5% SDS). The samples were then frozen at −20°C until used for DNA extraction.

#### 2.2.2. DNA Extraction

Samples were thawed and incubated overnight at 37°C with 100 ng/mL proteinase K (Sigma-Aldrich, St. Louis, MO, USA) under constant agitation. DNA was then purified as previously described [17]. DNA concentration in the final sample was estimated by absorbance at 260 nm, using a spectrophotometer (Thermo Fisher Scientific, Evolution 60, Madison, WI, USA).

#### 2.2.3. PCR

A 782 bp fragment of the intron 1 (+252A/G) of the LT-*α* gene was PCR-amplified with the primer set: (forward) 5′-AGA GGG GTG GAT GCT TGG GTT C-3′ and (reverse) 5′-CCG TGC TTC GTG CTT TGG ACT A-3′. PCR was carried out in a total volume of 10 *μ*L, containing 75 ng of genomic DNA, 0.1 *μ*M each primer and 1 U of Go Taq Green Master Mix (Promega Corporation, Madison, WI, USA). The solution was incubated for 5 min at 95°C, followed by 35 cycles of 1 min at 95°C, 1 min at 52°C, and 1 min at 72°C, with a final extension of 72°C for 7 min.

#### 2.2.4. Genotyping LT-*α* SNP rs909253

The PCR products were digested with 2U  *Nco*I per 25 *μ*L reaction volume at 37°C overnight. The digested fragments were separated by 10% polyacrylamide gel electrophoresis, and the gels were stained by the rapid silver staining method. The fragment containing the homozygous 2 allele (AA) of the LT-*α* gene was found to be not digested by *Nco*I and appeared as a single 782-bp band. The homozygous 1 allele (GG) is digested into 586-bp and 196-bp bands. All 3 fragments (782, 586, and 196 bp) are present in digested samples from the heterozygotes 1.2 (allele AG).

### 2.3. Statistical Analysis

Comparisons were made between allele (gene) and genotype frequencies in the periodontal disease and control populations. Allele frequencies were calculated from the observed numbers of genotypes. Differences in allele frequencies between the groups were determined by Fisher's exact test. Chi-squared analysis was used to test for deviation of genotype distribution from Hardy-Weinberg equilibrium and for a comparison of differences in genotype frequencies among groups. *P* values less than 0.05 were considered significant (BioEstat ver.5.0, Belém, PA, Brazil).

## 3. Results

The clinical parameters and differences between the patient and control groups are shown in Table 1. There were a higher proportion of women in the CP group than in the CG group, but this difference was not statistically significant (*P* = 0.0981).

**Distribution of the Inflammatory Cytokine LT-*α* SNP in CP and CG Groups**

We evaluated the Hardy-Weinberg equilibrium of the distribution of SNP genotypes for the patients and healthy individuals by the chi-squared test and found no significant difference between the 2 groups (*P* > 0.05).

The distribution of LT-*α* genotypes in CG and CP groups was significantly different (Table 2). The frequency of LT-*α* (+252) G/G and G/A was significantly higher in the CP group than in healthy controls (G/G + G/A versus AA; *P* = 0.0059, OR = 3.1, 95% CI = 1.45–6.65). When the allelic frequencies of the individual SNP were compared, the A allele was less frequently detected in the CP group than in the CG group (G versus A, *P* = 0.0019, OR = 2.67, 95% CI = 1.45–4.78). The proportion of individuals carrying the LT-*α* G/G genotype was significantly lower in the CG group (2%) than in the CP group (16%) (G/G + G/A versus A/A OR = 3.1, 95% CI = 1.45–6.65).

## 4. Discussion

Periodontitis is a multifactorial disease and is thought to result from a complex interaction between the dental biofilm and host immune system [18, 19]. Formation of the biofilm is necessary for periodontal disease to develop, although the absolute quantity and individual species of bacteria do not explain the complex process of the disease [20, 21]. Severe cases of periodontal disease may occur in the absence of a significant biofilm, suggesting that an exaggerated host inflammatory response makes an important contribution to the pathogenesis of the disease [20].

Several studies have shown a relationship between periodontal disease and genetic factors, including polymorphisms in interleukin (IL)-1 [22], interferon-*γ* [23], IL-6 [24], matrix metalloproteinase (MMP)-9, tissue inhibitor of MMP-2 [25], vitamin D receptor [26], IL-1*α*, and IL-1*β* [5].

Our data showed a significant association (*P* = 0.0019) between the LT-*α* (+252 A/G) polymorphism and CP in our Brazilian population. The G allele was more frequently observed in individuals with periodontal disease than in control subjects. The presence of only 1 G allele (G/A + G/G) in the genotype distribution of patients was sufficient to increase disease predisposition by a factor of 3 compared with individuals who had the A/A genotype. These data suggest that the G allele predisposes to periodontal disease. Interestingly, this has also been observed for LT-*α* (+252 A/G) in other diseases, including rheumatoid arthritis [10], asthma [11], cardiac diseases [12], lupus nephritis [27], breast cancer [28], and chronic Chagas cardiomyopathy [15].

The study of chronic Chagas cardiomyopathy patients found similar LT-*α* (+252 A/G) allelic frequencies as in the present study, which is to be expected because both investigations were performed in the Brazilian population. However, Fassmann and collaborators [14] investigated the correlation between periodontal disease and the same polymorphism LT-*α* (+252 A/G) in a Czech population, but the frequency of alleles in subjects with periodontitis and healthy individuals was different from those in our study [14]. These conflicting results may have been due to disease heterogeneity or the population heterogeneity. Smokers were excluded from our study but not from that conducted in the Czech population [14].

## 5. Conclusion

In summary, this study shows that the LT-*α* (+252 A/G) genetic polymorphism might be a risk indicator for susceptibility to chronic periodontal disease in the Brazilian population studied.

## Figures and Tables

**Table 1:** Clinical parameters of study population.

	Controls	Chronic periodontal disease
	*n* = 44	*n* = 82
Age (years)		
Mean ± SD (range)	39.0 ± 14.3	45.9 ± 10.3
Gender	(*P* = 0.0981)	
Male	27%	39%
Female	73%	61%
Pocket probing (mm)	1.9 ± 1.3	7.9 ± 1.9

**Table 2:** Frequencies of genotypes and alleles of polymorphism in LT-*α* (+252 A/G).

	Controls	Chronic periodontal disease
Genotypes	(*P* = 0.0059)	
LT-*α* GG	1 (2%)	13 (16%)
LT-*α* AG	17 (39%)	43 (52.5%)
LT-*α* AA	26 (59%)	26 (31.5%)
Allele frequencies	Odds ratio = 2.67, (*P* = 0.0019)	
Allele G (1)	0.22	0.42
Allele A (2)	0.78	0.58
